# Estimating the anomalous diffusion exponent for single particle tracking data with measurement errors - An alternative approach

**DOI:** 10.1038/srep11306

**Published:** 2015-06-11

**Authors:** Krzysztof Burnecki, Eldad Kepten, Yuval Garini, Grzegorz Sikora, Aleksander Weron

**Affiliations:** 1 Hugo Steinhaus Center, Wroclaw University of Technology, Wyspianskiego 27, 50-370 Wroclaw, Poland; 2 Physics Department & Institute for Nanotechnology, Bar Ilan University, Ramat Gan, 5290002, Israel

## Abstract

Accurately characterizing the anomalous diffusion of a tracer particle has become a central issue in biophysics. However, measurement errors raise difficulty in the characterization of single trajectories, which is usually performed through the time-averaged mean square displacement (TAMSD). In this paper, we study a fractionally integrated moving average (FIMA) process as an appropriate model for anomalous diffusion data with measurement errors. We compare FIMA and traditional TAMSD estimators for the anomalous diffusion exponent. The ability of the FIMA framework to characterize dynamics in a wide range of anomalous exponents and noise levels through the simulation of a toy model (fractional Brownian motion disturbed by Gaussian white noise) is discussed. Comparison to the TAMSD technique, shows that FIMA estimation is superior in many scenarios. This is expected to enable new measurement regimes for single particle tracking (SPT) experiments even in the presence of high measurement errors.

The field of biophysics and biomedicine has seen an immense increase in single particle tracking techniques and experimental results^1^,^2^. In the past decade, trajectories have been obtained for almost all biological entities, including *in vivo*^3^,^4^ and *in vitro*^5^ measurements that cover dynamics from the cell membrane^6^,^7^ to the nucleoplasm^8^,^9^. Biological trajectories are usually stochastic and affected by a great deal of randomness arising from thermal motion of surrounding molecules, spatial constraints, complex molecular interactions, water molecules on cell membranes and more^10^,^11^,^12^,^13^,^14^,^15^. While all these sources of stochasticity give rise to diffusive motion, each source has different characteristics, which can give important information regarding the biophysical system^16^.

The most popular theoretical models for the anomalous diffusion^17^ present in biophysical experiments are: continuous-time random walk (CTRW)^18^,^19^, obstructed diffusion (OD), fractional Brownian motion (FBM), autoregressive fractionally integrated moving average (ARFIMA), and fractional Langevin equation (FLE)^2^,^20^. These models can be divided into two categories: with short memory (CTRW, OD) and fractional with long (power-law) memory (FBM and ARFIMA). In this paper we concentrate on the latter class.

A common tool by which the anomalous diffusion of a single particle can be classified is the time-averaged mean square displacement (TAMSD):


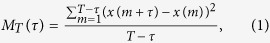


defined here for a trajectory *x*(*t*) of length *T* and the averaging window is 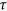. One of the fundamental properties is the scaling of TAMSD, i.e., 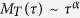. For normal diffusion the scaling is linear, *α* = 1, and anomalous diffusion shows a power law behaviour, with *α* > 1 termed superdiffusion and *α* < 1 called subdiffusion. The anomalous exponent *α* is connected to target finding times^21^, cellular organization^16^, reaction rates^22^ and more. In addition, the anomalous exponent can be connected to many other stochastic characteristics of the random walk, such as self similarity and long range correlations of displacements. Specifically, if we take an *H*-self-similar process with stationary and Gaussian increments, then *α* = 2*H* and the memory parameter *d* = *H *− 1/2^23^.

Unfortunately, the estimation of *α* for single trajectories is not a simple task. Usually the TAMSD is fitted to a power law – a method that is prone to estimation errors due to two main effects. The first arises from the fact that displacements in TAMSDs are not independent, and the central limit theorem does not work for a single trajectory^24^,^25^.

The second error arises from the inherent measurement error in any experimental procedure^26^. This has been shown to insert a bias towards lower *α* values at short times^27^. Thus fitting single particle TAMSDs results in anomalous exponents lower than the true physical process. It has been shown that this bias continues even to times where the TAMSD is larger than the measurement noise standard deviation. This effect cannot be corrected through ensemble averaging or measurement of longer trajectories and can be mitigated only under special conditions^28^.

Measurement errors, in the common stationary case, are a series of i.i.d. values added to the true location of the tracked particle. Thus the incremental process of the paricle is highly dependent between consecutive time points. If *δϵ*(*t*) and *δϵ*(*s*) are the increments of the error at time *t* and *s*, then they are strongly dependent for 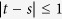 and independent otherwise. This leads to the possibility of separating the measurement error from the actual diffusion process, by distinguishing the transient short time correlation of the error from the long time correlation of the physical process.

We introduce the following toy model for experimental data with significant measurement noise. Let {*B*_*H*_(*t*), = 1,2,…,*T*} be a fractional Brownian motion (FBM) with the self-similarity index 0 < *H *< 1. This process will serve us as a basic model for a SPT. The choice of the FBM is well justified in the literature^2^,^12^,^13^,^29^,^30^,^31^,^32^. Now, we assume that a measurement error is given in the form of white Gaussian noise, namely i.i.d. random variables *ϵ*(*t*) with the normal distribution *N*(0, *σ*). As a consequence, the observed process is


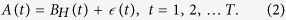


In all cases we take the variance of the increments of *B*_*H*_(*t*) to be equal to one. We also assume that *B*_*H*_(*t*) and ϵ**(*t*) are independent. Thus for this process 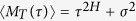 which deviates from the pure power law behaviour 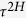 of the FBM. The difference can be clearly observed for small 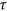
’s or large *σ*’s, and it influences the estimation of the anomalous exponent. It can also mimic the transient anomalous diffusion pattern. Moreover, the variance of the increments of *A*(*t*) is 1 + 2*σ*^2^. The autocorrelation function of the increments of *A*(*t*) equals


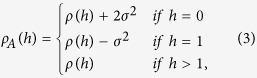


where *ρ*(*h*) is the autocorrelation function of the increments of FBM for a time lag *h*.

In this paper we investigate a new approach for the estimation of *α* under the presence of measurement errors. We approximate the toy model by the FIMA process which can be treated as a first order approximation of *ρ*_*A*_(*h*). This approximation takes into account the power law decay of the function controlled by the memory parameter *d* = *H* − 1/2 and the negative first lag correlation induced by the noise sequence, for *h* = 1 see equation (3). We also present a comprehensive comparison of the new estimator *α*_*FIMA*_ which is obtained by equation (11), with the classical estimator *α*_*TAMSD*_, given by equation (6), based on the time-averaged mean square displacement. To this end we analyze four representative cases of anomalous diffusion: *strong subdiffusion α* ∈ {0.4, 0.5, 0.6}, *weak subdiffusion α* ∈ {0.7, 0.8, 0.9}, *weak superdiffusion α* ∈ {1.1, 1.2, 1.3} and *strong superdiffusion α* ∈ {1.4, 1.5, 1.6}; and the case of the classical (normal) diffusion.

We compare the performance of the FIMA estimator to that of the common TAMSD for varying trajectory lengths, noise levels and all *α* regimes. The quality of the estimation of the anomalous exponent *α* is dependent on the the magnitude of the measurement error, *σ*, the length of the trajectory, *T*, and the value of the anomalous exponent itself. First, we study an influence of the noise parameter, *σ*, on the estimation error in Figs 1, 2, 3, 4, 5. To this end, we simulate a thousand of trajectories for *σ*’s from 0.25 to 2 with step 0.25 and *α*’s from 0.4 to 1.6 with step 0.1 and estimate *α* for each trajectory. The trajectories consist of *T* = 512 time points. Second, in Figs 6, 7, 8, 9, 10, we study an influence of the length of the trajectory, *T*, on the estimation error. To this end, we simulate a thousand of trajectories for *T* ∈ {256, 512, 1024, 2048} and *α*'s from 0.4 to 1.6 with step 0.1 and estimate *α* for each trajectory. The noise parameter *σ* is fixed and equal to 1. We find that the FIMA estimator is superior to the TAMSD in most cases.

We would also like to draw the attention of the reader to popular methods to determine *α* of single trajectories of FBM, namely the detrended fluctuation analysis (DFA)^33^ and detrending moving average (DMA) (34,35, where an interpretation of the possible intrinsic origin of the error in the moving average estimators is given in terms of an excess term in the Shannon entropy). For the extensive comparison of the DFA and DMA methods see^36^. In contrast, we concentrate here on the toy model *A*(*t*), which takes into account the measurement noise and propose the FIMA process as the appropriate approximation of the model.

## Results

This paper’s new topic is the extraction of anomalous diffusion exponent from raw single-particle trajectories for different anomalous diffusion regimes. The study’s benchmark is a comparison between the recovered parameters using the FIMA model and the classical TAMSD fit approach.

For both estimation techniques we calculate here the average estimated values: 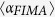 and 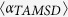, and the biases:


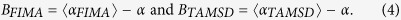


**Figure 1:**
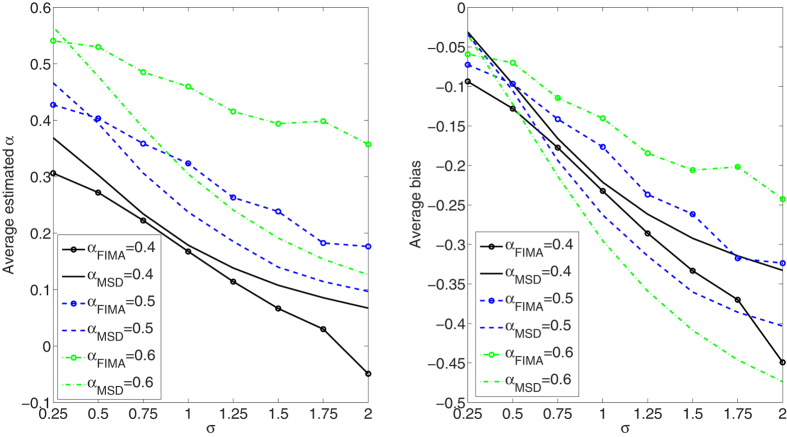
Strong subdiffusion case. Estimation of the anomalous exponent for different *σ*’s and *α*’s for 1000 trajectories of 2^9^ time points using the FIMA (marked with circles) and TAMSD frameworks. Average estimated *α* (left panel) and average bias (right panel).

**Figure 2:**
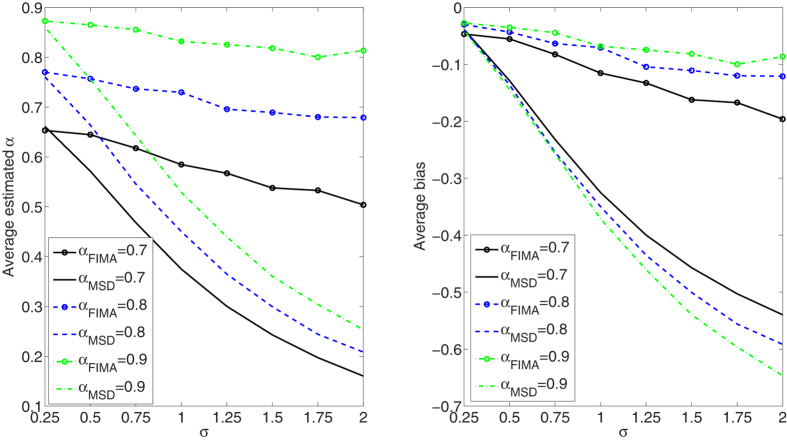
Weak subdiffusion case. Estimation of the anomalous exponent for different *σ*’s and *α*’s for 1000 trajectories of 2^9^ time points using the FIMA (marked with circles) and TAMSD frameworks. Average estimated *α* (left panel) and average bias (right panel).

**Figure 3:**
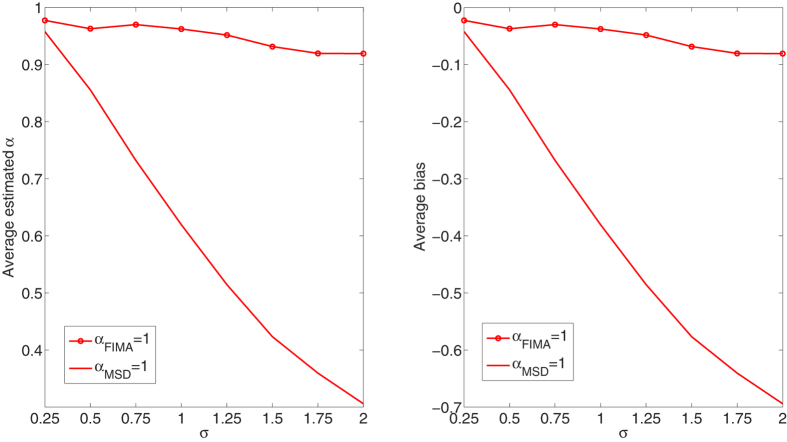
Classical diffusion case. Estimation of the anomalous exponent for different *σ*’s and *α* = 1 for 1000 trajectories of 2^9^ time points using the FIMA (marked with circles) and TAMSD frameworks. Average estimated *α* (left panel) and average bias (right panel).

**Figure 4:**
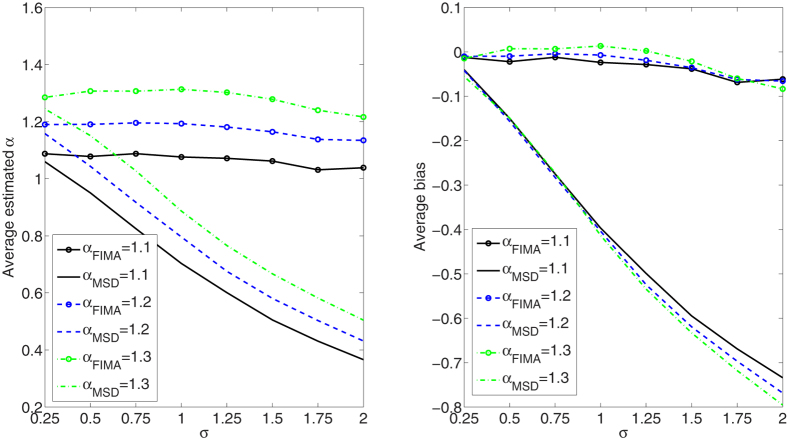
Weak superdiffusion case. Estimation of the anomalous exponent for different *σ*’s and *α*’s for 1000 trajectories of 2^9^ time points using the FIMA (marked with circles) and TAMSD frameworks. Average estimated *α* (left panel) and average bias (right panel).

**Figure 5:**
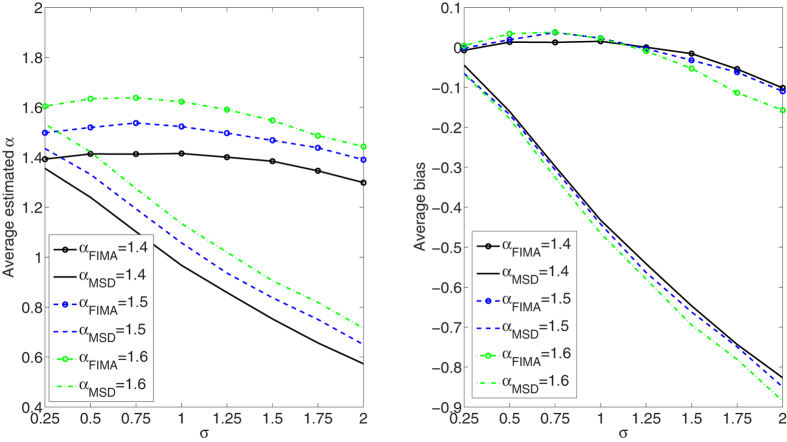
Strong superdiffusion case. Estimation of the anomalous exponent for different *σ*’s and *α*’s for 1000 trajectories of 2^9^ time points using the FIMA (marked with circles) and TAMSD frameworks. Average estimated *α* (left panel) and average bias (right panel).

In Figs 1, 2, 3, 4, 5 we depict the average estimated values and biases for *σ*’s from 0.25 to 2 with step 0.25 and *α*’s from 0.4 to 1.6 with step 0.1. To this end, we simulated a thousand of trajectories of *T* = 512 time points. First, let us notice that both methods often underestimate true values of the anomalous exponent. We can also observe that the FIMA estimator is superior to the TAMSD in the weak subdiffusion, diffusion and superdiffusion regimes. For the classical diffusion and superdiffusion cases the difference is striking for all *σ*’s, and growing rapidly with the measurement error. In the strong subdiffusion case for *α* = 0.4 TAMSD yields slightly better results. For *α* = 0.5 and *α* = 0.6 TAMSD gives more accurate estimates only for small sigmas, namely *σ* ≤ 0.6.

**Figure 6:**
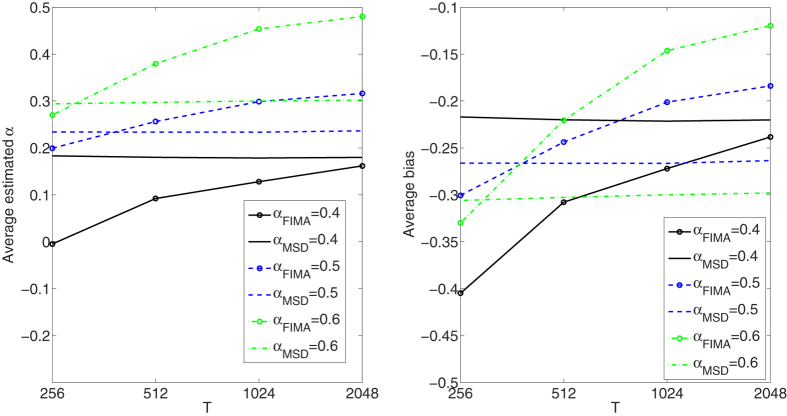
Strong subdiffusion case. Estimation of the anomalous exponent for *σ* = 1 and different *α*’s and trajectory lengths for 1000 trajectories using the FIMA (marked with circles) and TAMSD frameworks. Average estimated *α* (left panel). Average bias (right panel).

**Figure 7:**
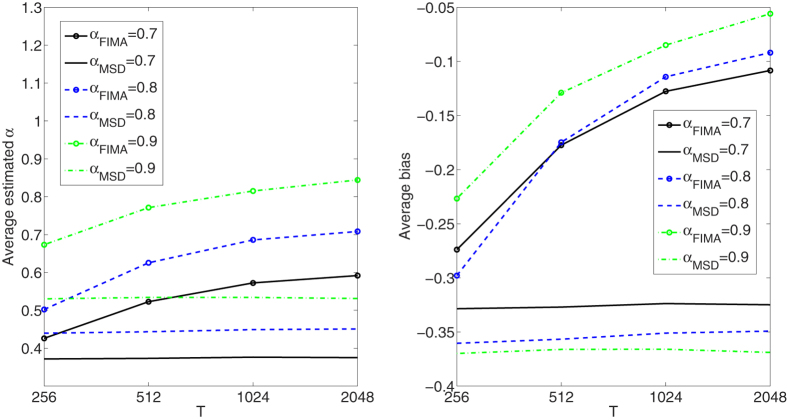
Weak subdiffusion case. Estimation of the anomalous exponent for *σ* = 1 and different *α*’s and trajectory lengths for 1000 trajectories using the FIMA (marked with circles) and TAMSD frameworks. Average estimated *α* (left panel). Average bias (right panel).

**Figure 8:**
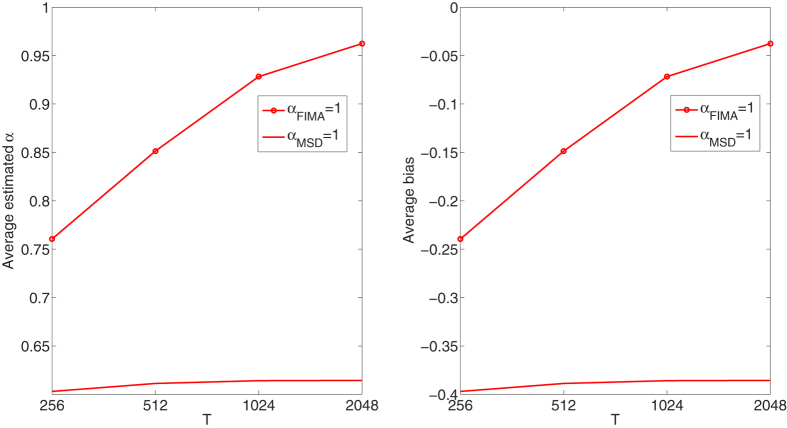
Classical diffusion case. Estimation of the anomalous exponent for *σ* = 1, *α* = 1 and different trajectory lengths for 1000 trajectories using the FIMA (marked with circles) and TAMSD frameworks. Average estimated *α* (left panel). Average bias (right panel).

**Figure 9:**
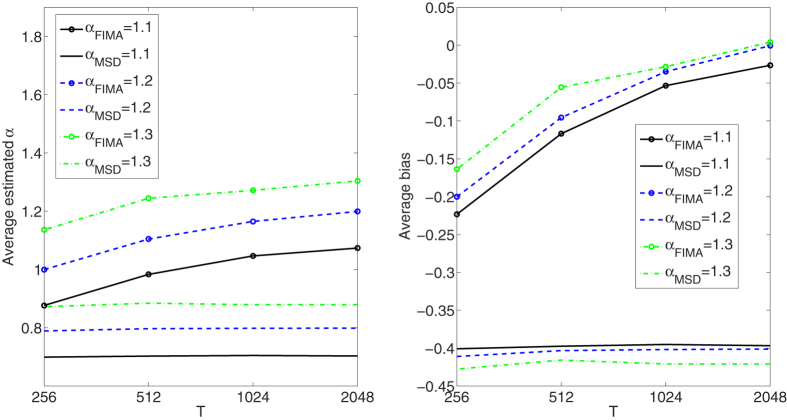
Weak superdiffusion case. Estimation of the anomalous exponent for *σ* = 1 and different *α*’s and trajectory lengths for 1000 trajectories using the FIMA (marked with circles) and TAMSD frameworks. Average estimated *α* (left panel). Average bias (right panel).

**Figure 10:**
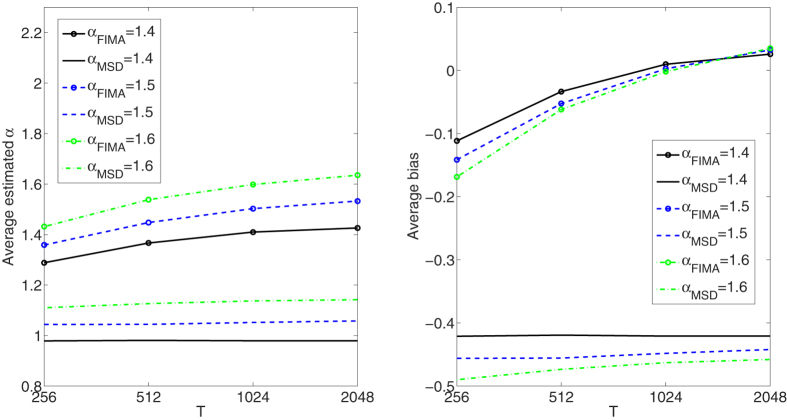
Strong superdiffusion case. Estimation of the anomalous exponent for *σ* = 1 and different *α*’s and trajectory lengths for 1000 trajectories using the FIMA (marked with circles) and TAMSD frameworks. Average estimated *α* (left panel). Average bias (right panel).

Next, in Figs 6, 7, 8, 9, 10 we present the effect of trajectory length on the results. We simulate a thousand of trajectories for *T* ∈ {256, 512, 1024, 2048} and *α*’s from 0.4 to 1.6 with step 0.1, estimating *α* for each trajectory. The noise parameter *σ* is fixed and equal to 1. For the strong subdiffusion case, for *α* = 0.4 TAMSD produces better results than the FIMA for all considered lengths of trajectories, but the difference between them is getting smaller as *T* grows. For *α* = 0.5 and *α* = 0.6 TAMSD yields better estimates only for *T* = 256. For the weak subdiffusion, classical diffusion and superdiffusion cases the FIMA estimator is always superior to the TAMSD and the differences become dramatic in the classical diffusion and superdiffusion regimes. Let us notice that the FIMA estimator, contrary to the TAMSD, clearly converges to the true values with increasing *T*.

We have also analyzed the relation between the magnitude of the measurement error and the moving average parameter 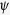 in both the subdiffusive (*α* = 0.8) and superdiffusive (*α* = 1.2) domains. The results are presented in Supplementary Fig. S1. For both anomalous cases with increasing variance of *ϵ*(*t*) the coefficient 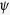 grows monotonously. Therefore, we may claim that the moving average part contains the information about the measurement error. In the future, it may be possible to conclude the magnitude of the measurement error from 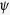 without experimental calibration.

Finally, in the supplementary material we provide an extensive statistical analysis of the estimators in the form of box plots, see Supplementary Figs S2–S11. This statistical analysis confirms the previous findings from Figs 1, 2, 3, 4, 5, 6, 7, 8, 9, 10. In particular, the range between whiskers in box plots provides information about variability in a distribution of the estimator. We can see that the variance of the TAMSD estimator is lower than that of FIMA in many cases, which is especially visible in the strong subdiffusion regime. Moreover, in this regime the variance of the FIMA estimator grows as *σ* increases. The situation changes as we proceed to the weak subdiffusion and further to superdiffusion regimes. In the weak subdiffusion case, the variance of the introduced estimator becomes comparable to the TAMSD for small *σ*’s. For the superdiffusion regimes, the variance of the FIMA estimator is comparable to the TAMSD’s for almost all possible *σ*’s.

While the variance of the TAMSD estimator is lower than that of FIMA in many cases, the large bias deems it inaccurate. In many cases, the quartile intervals for TAMSD and FIMA are even disjoint, which statistically disqualifies the TAMSD method.

## Discussion

In this paper we showed how to apply an anomalous diffusion exponent estimation algorithm based on the FIMA model. For a toy model representing a typical measurement, we compared the FIMA estimation results with those obtained by the popular TAMSD estimation.

The FIMA model is a special case of the ARFIMA process^37^,^38^,^39^,^40^,^41^ (the acronyms “ARFIMA” and “FARIMA” are often used interchangeably in the literature) which, from the physical point of view, is a discrete time analogue of the fractional Langevin equation that takes into account the memory parameter *d*^42^. ARFIMA have been already studied in the physical literature^43^,^44^,^45^,^46^,^47^,^48^,^49^,^50^. However, interpretation of the various parameters in an experimental context, to the best of our knowledge, is still missing.

The main finding of this paper is that the FIMA approach leads to more accurate values of the anomalous exponent in SPT experiments than by using standard TAMSD data fitting. This was confirmed for trajectories with *α* ≥ 0.5, *σ* ≥ 0.5 and *T* ≥ 512, see Figs 1, 2, 3, 4, 5, 6, 7, 8, 9, 10. The idea put forward in the paper is that the FI part of the process gives rise to long memory effects, while the MA part mimics the short memory effects that appear due to measurement errors. As a consequence the FIMA(*d*,1) model can identify measurement errors in such experiments. The estimated parameters *α* and 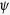 provide information about the magnitude of the error, see Supplementary Fig. S1. Identification of the measurement error magnitude can be realized by a calibration surface which will be discussed elsewhere.

We showed that the FIMA framework can extract accurate *α* values even under high measurement error with smaller bias than the common TAMSD technique. It allows a richer modelling scheme than other common models such as FBM^29^, once the physical interpretation of the parameters is understood. The analysis of stochastic trajectories below the error threshold, reduces the experimental limitations on particle localization, enabling the measurement of biophysical trajectories in faster frame rates and longer trajectories. Moreover, our methodology can be extended to physical and biological systems described by Lévy stable distribution^23^,^51^.

Finally, we find that the ARFIMA framework is a promising tool in the analysis of anomalous diffusion processes, especially when physical intuition is coupled to its mathematical components. From an experimental point of view, the FIMA model enables more accurate analysis of trajectories with higher measurement error levels without the need for calibration. This in turn is expected to enable longer and faster measurements and hopefully the study of new phenomena and biophysical entities.

## Methods

### TAMSD estimation algorithm

If the trajectory comes from a FBM, then


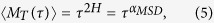


and consequently *α*_*MSD*_ = 2*H*. For the Brownian motion (*H* = 1/2) we arrive at the diffusion case, namely 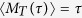. If *H* < 1/2, so in the negative dependence case, the process follows the subdiffusive dynamics, if *H* > 1/2, the character of the process changes to superdiffusive.

Hence, in order to estimate the anomalous diffusion exponent *α*, we calculate the following equation:


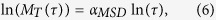


where 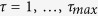 and 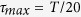. For other possibilities see^52^.

### FIMA framework

The field of econometrics has a long history of analyzing random motion in order to extract controlling parameters and predicting future behaviour^37^,^38^,^39^. While many of the mathematical models and their basic approach are different than what is common in physical or biological sciences, it is worthwhile to look upon them as a source for novel techniques. In this paper, we study a fractionally integrated moving average FIMA(*d*,*q*) process *X*(*t*), which is a special case of the general ARFIMA(*p*,*d*,*q*) process^38^,^40^,^41^. FIMA is represented by the fractional difference equation:


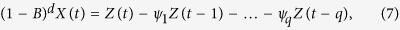


where *t* = 0,±1,…, and *B* is the shift operator: *BX*(*t*) = *X*(*t* − 1). In addition −1/2 < *d* < 1/2, taking fractional values, either positive or negative, and {*Z*(*t*)} is a white noise sequence^53^.

The fractional difference operator (1 − *B*)^*d*^ is defined by means of the binomial expansion, namely 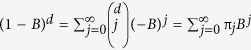, where 
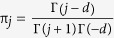
 and Γ is the Gamma function. The polynomial 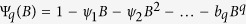 has no roots in the closed unit disk. It corresponds to the moving average (MA) part.

Hence, the model combines two broad classes of time series, namely the fractionally integrated (FI) models, and the moving average (MA) models^40^,^41^,^54^. The latter reconstructs a short-term memory structure given by the autocorrelation function for short lags, whereas the former a power-law behaviour for large lags, which leads to the notion of long-term dependence. The MA part provides also a mechanism of transformation of uncorrelated inputs to correlated outputs in many physical systems, see^55^,^56^.

In many applications FIMA(*d*,1) model is sufficient to describe the data well, see, e.g.^44^. FIMA(*d*,1) can be considered a first-order approximation of the arbitrary short memory structure (*q* lags) taking into account only the first lag. In this case the model reduces to:


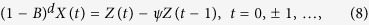


The basic building block of FIMA(*d*,1) model is the MA(1) process: 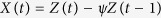, which is a special case of the MA(*q*) model^40^,^41^. It appears that if *X*(*t*) is a stationary 1-correlated time series, i.e., *X*(*s*) and *X*(*t*) are independent whenever 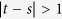 (in contrast to an i.i.d. sequence, which is zero-dependent), then it can be represented as the MA(1) process^40^,^41^. The dependence is only one lag long and it’s intensity is fully controlled by the parameter 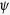. Hence, the MA model introduces a short memory of the process. In general, the MA(*q*) process may reconstruct any arbitrary short time (finite lag) correlation structure from the experimental data. The fractional integration introduces the long (power-law) memory, which is defined by the memory parameter *d*. The FIMA(*d*,1) process is well-defined for −1/2 < *d* < 1/2. Such processes are asymptotically *H*-self-similar with the parameter *H* = *d* + 1/2. The rate of decay of the autocovariance function of the FIMA(*d*,1) model is


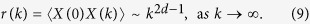


Therefore, for *d* > 0 we have 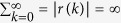. This serves as a classical definition of long memory and is equivalent to the case of FBM with *H* > 1/2. Similarly, for *d* < 0 we arrive at the negative power law decay, which corresponds to FBM with *H* < 1/2. The case *d* = 0 leads to the moving average (MA) model, which has exponentially decaying autocorrelation function^40^,^41^,^43^.

Furthermore, Brownian motion (BM) corresponds, in the limit sense^57^, to FIMA(0,0). Similarly, FBM corresponds to FIMA(*d*,0) with *d* = *H* − 1/2, where *H* is the self-similarity parameter. The FIMA processes offer flexibility in modelling long power-law and one-lag dependencies by choosing the memory parameter *d* and the appropriate moving average coefficient 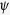 in equation (8). Hence, it is possible to model and characterize more complex processes than using FBM alone.

### FIMA estimation algorithm

To make the FIMA(*d*,1) model feasible in applications, we need an efficient estimator of its parameters. Modifying^58^,^59^, we estimate the vector 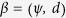. For a sample {*x*_1_,*x*_2_,…,*x*_*N*_} we denote the normalized periodogram by


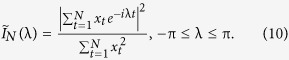


The estimator of the parameter vector *β* is defined as the vector argument 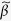, for which the following function attains its minimum value:


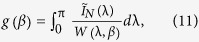


where


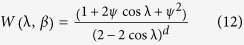


is the spectral density of the FIMA process. The idea of the estimator is to find a parameter vector 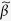 for which the spectral density 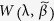 is the closest to its empirical counterpart, namely the periodogram 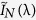. Such vector minimizes the value of the integral. The idea is similar to the standard maximum likelihood technique. In order to calculate 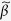, we have used *fminsearch* function implemented in Matlab, which applies the simplex search method of^60^.

## Additional Information

**How to cite this article**: Burnecki, K. *et al.* Estimating the anomalous diffusion exponent for single particle tracking data with measurement errors - An alternative approach. *Sci. Rep.* **5**, 11306; doi: 10.1038/srep11306 (2015).

## Supplementary Material

Supplementary Information
