# Supplementary material for: Estimating the anomalous diffusion exponent for single particle tracking data with measurement errors - An alternative approach
Source: Sci Rep. 2015 Jun 11;5:11306. doi: 10.1038/srep11306 (PMC4463942; doi:10.1038/srep11306)
Supplement: Supplementary Information [file srep11306-s1.pdf]

Supplementary information

Estimating the anomalous diffusion exponent for single particle tracking data with measurement errors - An alternative approach

Krzysztof Burnecki[1], Eldad Kepten[2], Yuval Garini[2], Grzegorz Sikora[1] & Aleksander Weron[1]

1. Hugo Steinhaus Center, Wroclaw University of Technology, Wyspianskiego 27, 50-370 Wroclaw, Poland
2. Physics Department & Institute for Nanotechnology, Bar Ilan University, Ramat Gan, Israel

I. MONOTONICITY OF DEPENDENCE BETWEEN THE MAGNITUDE OF THE MEASUREMENT ERROR AND THE MOVING AVERAGE PARAMETER

In Fig. S1 we illustrate the relation between the magnitude of the measurement error and the moving average parameter ψ in both the subdiffusive ($\alpha = 0.8$) and superdiffusive ($\alpha = 1.2$) domains. We can observe that for both cases with increasing variance of $\epsilon(t)$ the coefficient ψ grows. This leads to the conclusion that the moving average part of the FIMA process contains the information about the measurement error.

II. BOXPLOTS OF THE ESTIMATED ANOMALOUS EXPONENTS FOR DIFFERENT σ'S

We present now the statistical analysis of the accuracy of estimators for the toy model in terms of box plots. The box has lines at the lower quartile, median, and upper quartile values. The whiskers are lines extending from each end of the box to show the extent of the rest of the data. They correspond to the confidence interval covering approximately 99% of the data.

In Figs. S2-S6 we depict box plots of estimated values of the anomalous exponents obtained by FIMA and MSD techniques. Each box plot is constructed from a thousand of trajectories of $T = 1024$ time points. The parameter σ changes from 0.25 to 2 with step 0.25 and α from 0.4 to 1.6 with step 0.1. We notice that both methods are prone to underestimation of the true values of the anomalous exponent. We can also observe that the FIMA estimator is superior to the MSD in the weak subdiffusion, diffusion and superdiffusion regimes. For the classical diffusion and superdiffusion cases the difference becomes striking as σ grows. Only in the strong subdiffusion case for $\alpha = 0.4$ MSD produces slightly more accurate results. For $\alpha = 0.5$ and $\alpha = 0.6$ MSD works better only for small sigmas, namely $\sigma \leq 0.6$.

We can also observe see that the variance of the MSD estimator is usually lower than of the FIMA. However, the accuracy of the FIMA estimator is clearly demonstrated in the above mentioned cases. We can notice, that for some cases the quartile intervals of the two estimators are even disjoint.

III. BOXPLOTS OF THE ESTIMATED ANOMALOUS EXPONENTS FOR DIFFERENT TRAJECTORY LENGTHS

In Figs. S7-S11 we depict box plots of estimated values of the anomalous exponents obtained by FIMA and MSD techniques. Each box plot is constructed from a thousand of trajectories of $T = 256, 512, 1024$ and 2048 time points. The parameter α changes from 0.4 to 1.6 with step 0.1 and σ is fixed and equal to 1. We can observe that in the strong subdiffusion regime the MSD estimator is better than the FIMA only for the smallest trajectory length, namely for $T = 256$. For other regimes the FIMA technique is always superior. We can also notice that the FIMA estimator converges to the true value of α as the trajectories become longer. This suggests the consistency of the FIMA estimator.

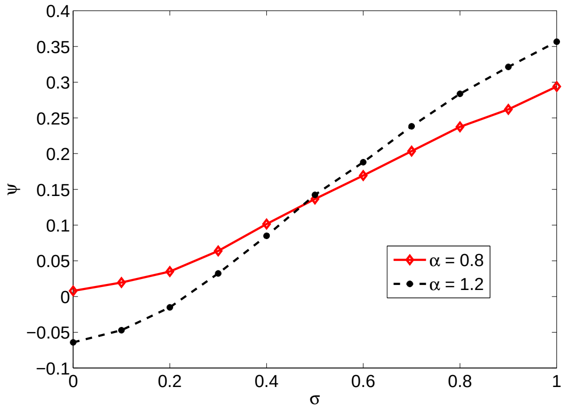

FIG. S1: Estimation of the moving average coefficient ψ for $\alpha = 0.8$ (red diamonds) and $\alpha = 1.2$ (black points) for different σ's ($\sigma = 0$ corresponds to $B_H(t)$). We can clearly observe that the moving average part of FIMA grows as the variance of the measurement error increases.

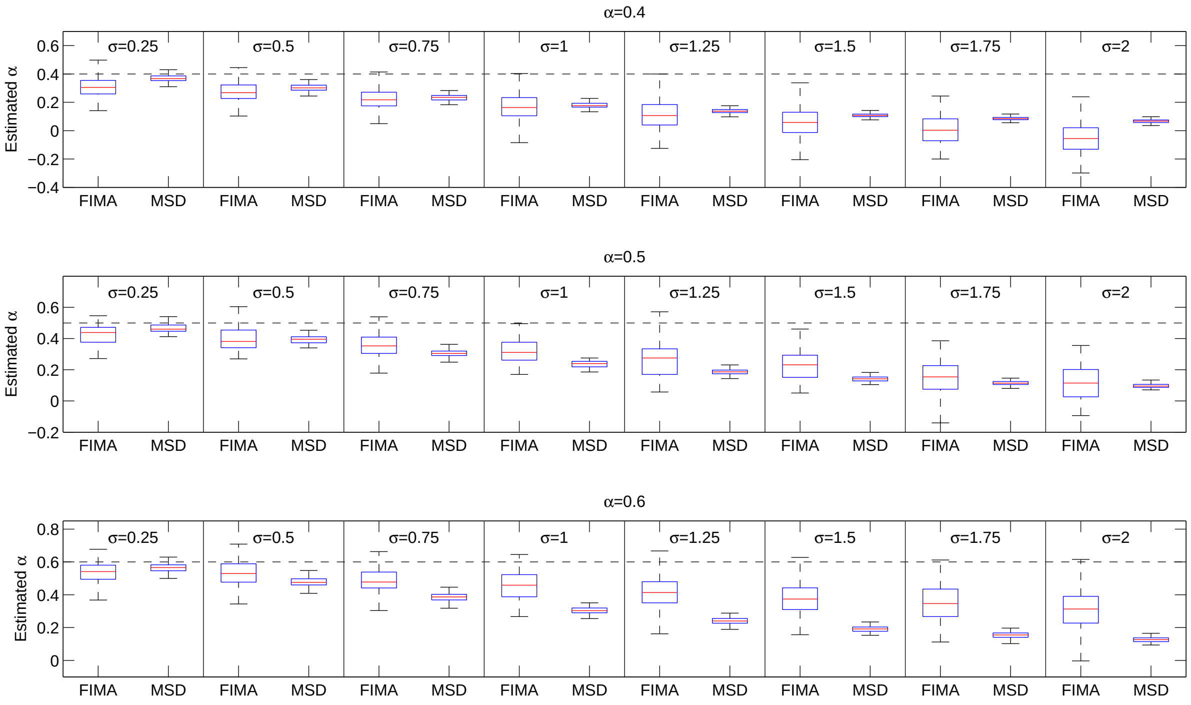

FIG. S2: Boxplots of the estimated anomalous exponent for strong subdiffusion case and different σ. For each case, 1000 trajectories of length $T = 1024$ were simulated.

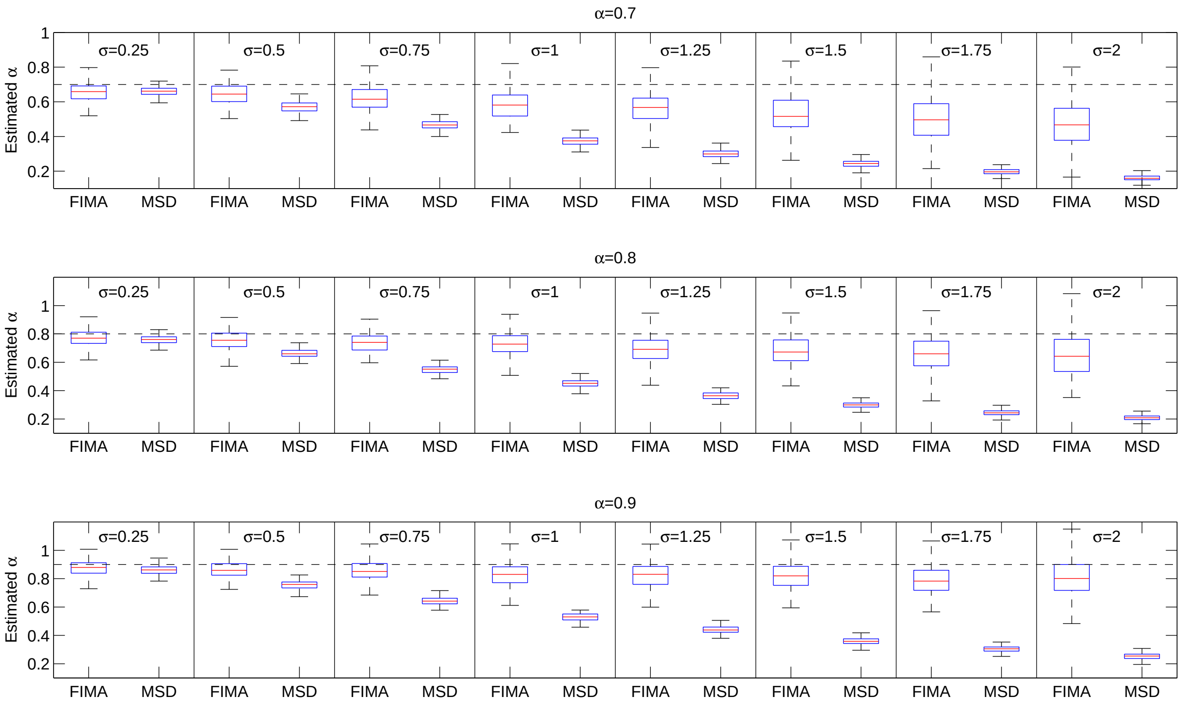

FIG. S3: Boxplots of the estimated anomalous exponent for weak subdiffusion case and different σ. For each case, 1000 trajectories of length $T = 1024$ were simulated.

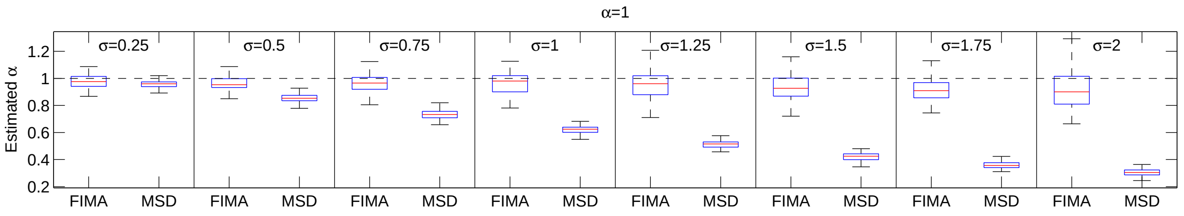

FIG. S4: Boxplots of the estimated anomalous exponent for pure diffusion case with $\alpha = 1$ and different σ. For each case, 1000 trajectories of length $T = 1024$ were simulated.

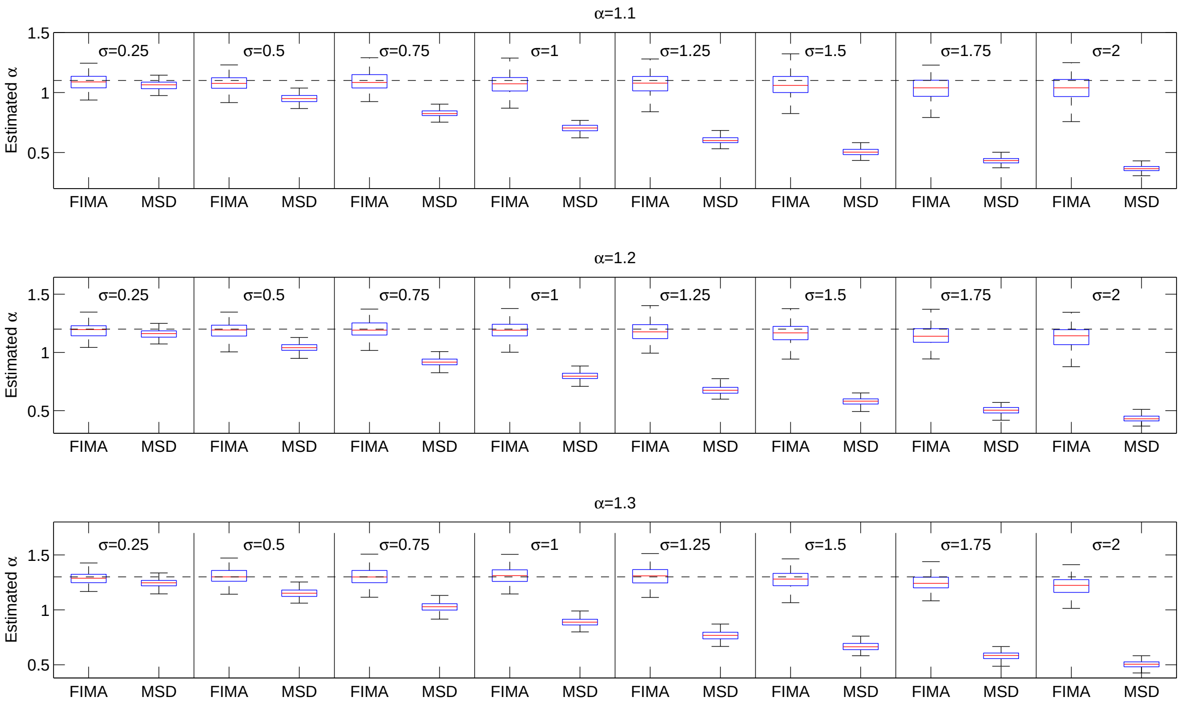

FIG. S5: Boxplots of the estimated anomalous exponent for weak superdiffusion case and different σ. For each case, 1000 trajectories of length $T = 1024$ were simulated.

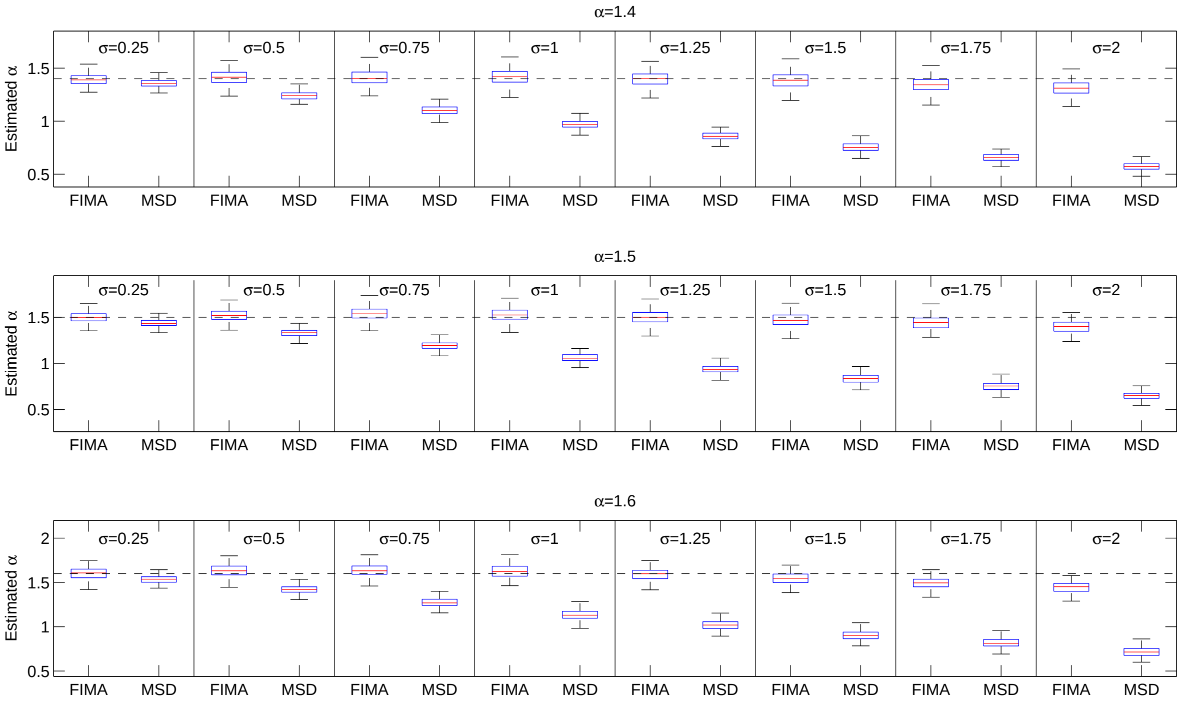

FIG. S6: Boxplots of the estimated anomalous exponent for strong superdiffusion case and different σ. For each case, 1000 trajectories of length $T = 1024$ were simulated.

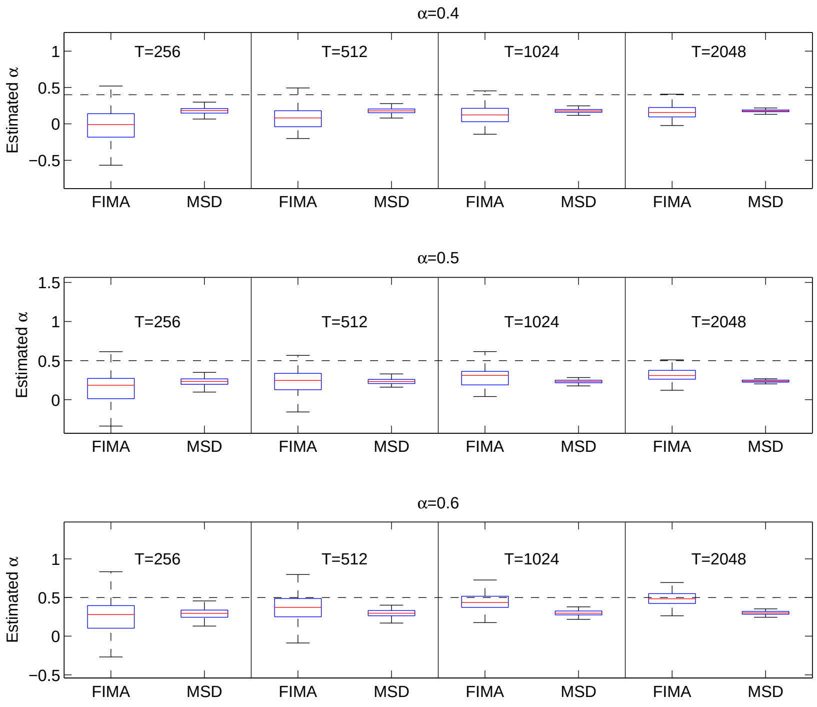

FIG. S7: Boxplots of the estimated anomalous exponent for strong subdiffusion case, different trajectory length and $\sigma = 1$. 1000 trajectories were simulated for each case.

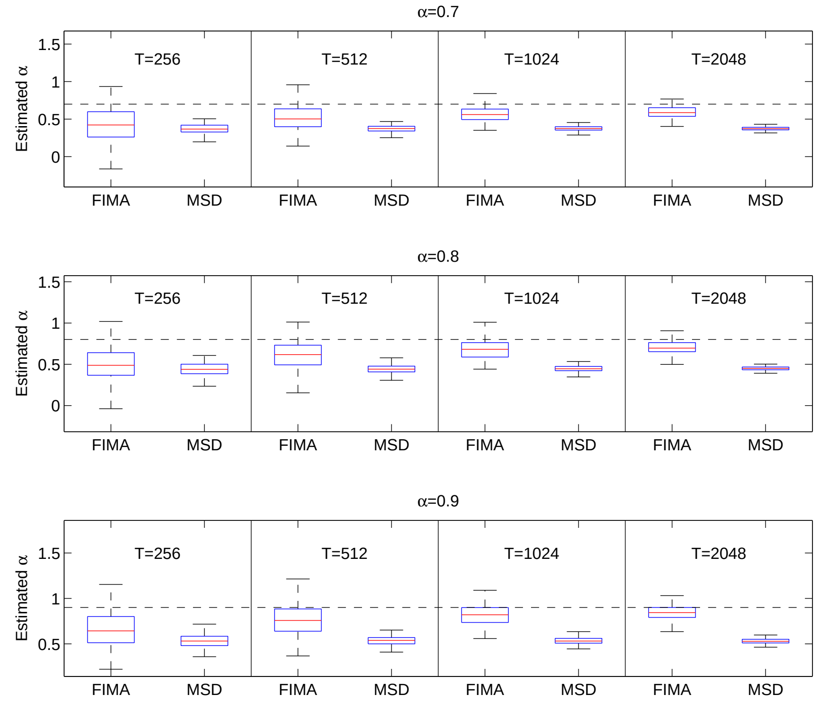

FIG. S8: Boxplots of the estimated anomalous exponent for weak subdiffusion case, different trajectory length and $\sigma = 1$. 1000 trajectories were simulated for each case.

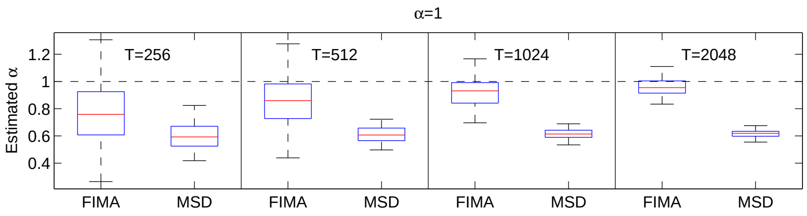

FIG. S9: Boxplots of the estimated anomalous exponent for pure diffusion case with $\alpha = 1$, different trajectory length and $\sigma = 1$. 1000 trajectories were simulated for each case.

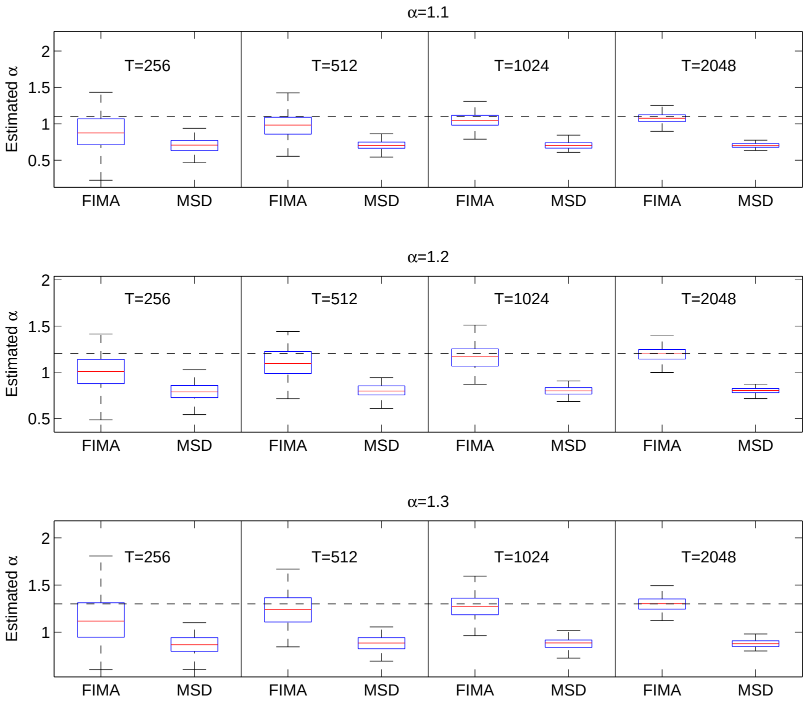

FIG. S10: Boxplots of the estimated anomalous exponent for weak superdiffusion case, different trajectory length and $\sigma = 1$. 1000 trajectories were simulated for each case.

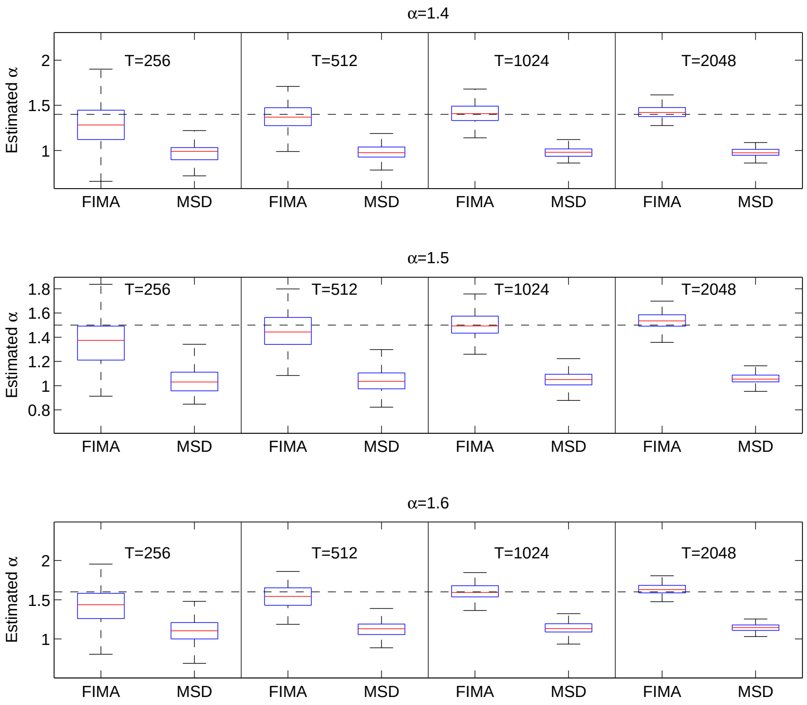

FIG. S11: Boxplots of the estimated anomalous exponent for strong superdiffusion case, different trajectory length and $\sigma = 1$. 1000 trajectories were simulated for each case.
